# Estrogen Receptor α36 Mediates a Bone-Sparing Effect of 17β-Estrodiol in Postmenopausal Women

**DOI:** 10.1002/jbmr.169

**Published:** 2010-06-24

**Authors:** Hui Xie, Mei Sun, Xiao-Bo Liao, Ling-Qing Yuan, Zhi-Feng Sheng, Ji-Cai Meng, Dan Wang, Zhi-Yong Yu, Lei-Yi Zhang, Hou-De Zhou, Xiang-Hang Luo, Hui Li, Xian-Ping Wu, Qi-You Wei, Si-Yuan Tang, Zhao-Yi Wang, Er-Yuan Liao

**Affiliations:** 1 Institute of Endocrinology and Metabolism, Second Xiangya Hospital of Central South UniversityChangsha, Hunan, People's Republic of China; 2 Department of Pathology, Second Xiangya Hospital of Central South UniversityChangsha, Hunan, People's Republic of China; 3 Department of Spine Surgery, Second Xiangya Hospital of Central South UniversityChangsha, Hunan, People's Republic of China; 4 Department of Cardiothoracic Surgery, Second Xiangya Hospital of Central South UniversityChangsha, Hunan, People's Republic of China; 5 School of Nursing of Central South UniversityChangsha, Hunan, People's Republic of China; 6 Department of Medical Microbiology and Immunology, Creighton University Medical SchoolOmaha, NE. USA

**Keywords:** ESTROGEN RECEPTOR α36, OSTEOBLAST, OSTEOCLAST, APOPTOSIS, BONE MINERAL DENSITY

## Abstract

Recently, a membrane-based estrogen receptor (ER), ER-α36, was identified and cloned that transduces membrane-initiated estrogen signaling such as activation of the mitogen-activated protein kinase/extracellular signal-regulated kinase (MAPK/ERK) signaling pathway. Here we show that the postmenopausal level of estradiol (E2) induces mitogenic, antiapoptotic, and antiosteogenic effects and proapoptotic effects in postmenopausal osteoblasts and osteoclasts with high levels of ER-α36 expression, respectively. We also found that ER-α36 mediated the effects of postmenopausal-level E_2_ on proliferation, apoptosis, and differentiation of osteoblasts through transient activation of the MAPK/ERK pathway, whereas ER-α36-mediated postmenopausal-level E_2_ induces apoptosis of osteoclasts through prolonged activation of the MAPK/ERK pathway with the involvement of reactive oxygen species. We also show that the levels of ER-α36 expression in bone are positively associated with bone mineral density but negatively associated with bone biochemical markers in postmenopausal women. Thus the higher levels of ER-α36 expression are required for preserving bone mass in postmenopausal and menopausal women who become osteoporotic if ER-α36-mediated activities are dysregulated. © 2011 American Society for Bone and Mineral Research.

## Introduction

Estrogen triggers two different modes of signal transduction: classic genomic actions and rapid nongenomic actions that rely on cytoplasmic signal-transduction cascades.(1,2) Nongenomic estrogen signaling plays an important role in bone protection.(3–5) Estrogen is able to protect the adult skeleton against bone loss by maintaining a focal balance between bone formation and resorption, which mainly results from the opposite effects of osteoblasts (OBs) and osteoclasts (OCs). Estrogen has antiapoptotic effects on OBs and proapoptotic effects on OCs(6–8) through an extranuclear signaling that leads to activation of the mitogen-activated protein kinase/extracellular signal-regulated kinase (MAPK/ERK) signaling pathway and kinase-dependent changes in transcription activities.(4,5,9) It has been postulated that the kinetics of ERK phosphorylation and the length of time that phospho-ERKs are retained in the cell nucleus determine the pro- or antiapoptotic effects of estrogen(9); the transient activation of the MAPK/ERK pathway induces antiapoptotic effects, whereas prolonged ERK activation is associated with proapoptotic effects.

Estrogen receptors (ER-α, its splice variant ER-α46, and ER-β) are defined as ligand-activated transcription factors that regulate transcription of estrogen-responsive genes in the cell nucleus.(10,11) ER-α36, a recently identified variant of ER-α (ER-α66), is generated from a previously unidentified promoter located in the first intron of the *ERα66* gene.(12) It lacks both transcriptional activation domains (AF-1 and AF-2) of *ERα66*, retains the DNA-binding and dimerization domains, and has a partial ligand-binding domain. In addition, it possesses a unique 27-amino-acid domain to replace the last 138 amino acids encoded by exons 7 and 8 of the *ERα66* gene. Recent studies have shown that ER-α36 is expressed in specimens from breast cancer patients, established breast cancer cell lines,(13,14) endometrial cancer cells,(15) colorectal cancers cells,(16) and mouse ovaries.(17) Unlike ER-α66, which is often expressed in the cell nucleus and mediates genomic estrogen signaling, ER-α36 localizes on the plasma membrane and elicits the membrane-initiated estrogen signaling.(12,13) Recently, we found that cells expressing high levels of ER-α36 are hypersensitive to E_2_, activating the MAPK/ERK pathway in the picomolar range.(18)

In this study we observed high levels of ER-α36 expression in OBs and OCs from normal postmenopausal women and assessed its role in postmenopausal low-level E_2_ -mediated mitogenic, antiapoptotic, and antiosteogenic effects in OBs and proapoptotic effects in OCs. We also analyzed the correlation coefficients between ER-α36 expression in bone and bone mineral density (BMD) and the serum bone biochemical markers in pre- and postmenopausal women.

## Materials and Methods

### Study population

The clinical study was approved by the Ethics Committee of the Second Xiangya Hospital of Central South University, and written informed consent was obtained from all participants. The study population consisted of 154 Chinese women (premenopausal: 60; postmenopausal: 33 normal, 31 osteopenic, and 30 osteoporotic) who underwent surgery for intervertebral disk hernia, spinal stenosis, or spondylolisthesis at the Second Xiangya Hospital of Central South University from 2006 to 2009. In order to select the study population, 460 postmenopausal women and 92 premenopausal women who underwent surgery for intervertebral disk hernia, spinal stenosis, or spondylolisthesis were screened for BMD and E_2_ levels; all 552 subjects had cancellous bone explants. All subjects were screened with a detailed questionnaire, medical history, and physical examination before surgery. Subjects were excluded from the study if they had conditions that affect bone metabolism, including diseases of the kidney, liver, parathyroid, and thyroid, or any of the following conditions: diabetes mellitus, hyperprolactinemia, oophorectomy, rheumatoid arthritis, ankylosing spondylitis, malabsorption syndromes, malignant tumors, hematologic diseases, or previous pathologic fractures. Other medical conditions for which subjects were excluded from the study were hypertension, chronic liver disease, coronary artery disease, angiopathy, myocardial infarction, cerebral infarction, and infectious disease. If the subjects had received treatment with glucocorticoids, estrogens, thyroid hormone, parathyroid hormone, fluoride, bisphosphonate, calcitonin, thiazide diuretics, barbiturates, or antiseizure medication, they also were excluded. Body weight was measured using a standardized balance-beam scale.

### BMD measurement

BMD was measured using a dual-energy X-ray absorptiometry (DXA) fan-beam bone densitometer (Hologic QDR 4500A, Hologic, Inc., Bedford, MA, USA) at the lumbar spine (L_1_ –L_4_ ) and the left hip as described previously by our group.(19–21) All BMD results are expressed in grams per square centimeter (g/cm^2^). The control spine phantom scan performed each day had a long-term (>10 years) coefficient of variation of less than 0.43%. According to the World Health Organization definition(22) and the BMD reference databases established by our group,(19,20) subjects with a BMD of 2.5 SDs lower than the peak mean of the same gender (*T*-score ≤ −2.5) were determined to be osteoporotic, those with a *T*-score between −1.0 and −2.5 were determined to be osteopenic, and those with a *T*-score > − 1.0 were determined to be normal.

### Measurement of serum E_2_, sex hormone–binding globulin (SHBG), and bone biochemical markers

Blood samples were collected between 7:00 to 9:00 a.m. after overnight fasting, and the samples were allowed to clot at room temperature. The samples then were centrifuged, divided into aliquots, and stored at −80°C until use. Radioimmunoassay was employed to measure serum E_2_ (Diagnostic Products Corp., Deerfield, IL, USA) and SHBG (Diagnostic Systems Laboratories, Webster TX, USA). The lower limit of detection was 5.1 pM for E_2_ and 3 nM for SHBG, respectively. The intra- and interassay coefficients of variation were 7.6% and 8.2% for E_2_ and 4.9% and 6.6% for SHBG, respectively. The free estrogen index (FEI) was calculated as the molar ratio of total E_2_ to SHBG. ELISA was used to measure serum osteocalcin (Diagnostic Systems Laboratories) and cross-linked *N*-telopeptides of type I collagen (NTX; Osteomark, Ostex International Inc., Princeton, NJ, USA). The intra- and interassay coefficients of variation were 4.2% and 5.3% for osteocalcin and 6.3% and 7.1% for NTX, respectively. The absorbance was recorded on a µQuant microplate spectrophotometer (Bio-Tek Instruments, Inc., Winooski, VT, USA).

### Bone sample collection

Vertebral cancellous or cortical bone explants were obtained from women who underwent surgery for intervertebral disk hernia, spinal stenosis, or spondylolisthesis. Limb cancellous and cortical bone samples were obtained during surgery from victims of road traffic accidents. Every vertebral cancellous bone sample was snap frozen in liquid nitrogen and stored at –80°C for RNA isolation. Vertebral and limb bone explants also were fixed in formaldehyde and then decalcified in an HCl–formic acid solution, followed by dehydration in graded ethanol and embedding in paraffin. All subjects were provided informed consent before surgical operation.

### Immunohistochemistry

After deparaffinization, immunohistochemical staining of ER-α36 in bone sections was performed as described previously.(23) Briefly, the slides were incubated with the primary antibody (a polyclonal rabbit anti-ER-α36 antibody raised against the last 20 amino acids as a custom service by Alpha Diagnostic International (San Antonio, TX, USA; dilution 1:50), as described previously,(13) and stored overnight at 4°C. Biotinylated goat antirabbit secondary antibody (1:100; Santa Cruz Biotechnology, Inc., Santa Cruz, CA, USA) then was added and incubated for 30 minutes at room temperature. 3-Amino-9-ethylcarbazole was used as the chromogen for 5 minutes.

### Bone tissue and cell culture

Vertebral cancellous bone explants were cultured in phenol red–free α modified essential medium (α-MEM, Sigma, St Louis, MO, USA) containing 10% charcoal/dextran-treated FBS (HyClone, Logan, UT, USA), 100 U/mL of penicillin, 100 µg/mL of streptomycin, and 50 µg/mL of ascorbic acid (Sigma) at 37°C.

Primary OBs and OCs were isolated from human vertebral bone as described previously.(21,24,25) Briefly, vertebral cancellous bone explants were thoroughly rinsed with serum-free α-MEM and digested with type IV collagenase (Sigma). Cells were cultured in phenol red–free α-MEM containing 10% charcoal/dextran-treated FBS. These primary OBs were characterized as described previously by our group.(21,26) Bone marrow cells were prepared from vertebral cancellous bone explants. Primary OBs (2 × 10^4^ cells/well) and bone marrow cells (2 × 10^5^ cells/well) were cocultured in 24-well plates containing phenol red–free α-MEM with 10% charcoal/dextran-treated FBS and 10 nM 1,25-dihydroxyvitamine D_3_ [1,25(OH)_2_ D_3_ ] for 7 to 11 days with a change of medium every 2 days. After 7 days in culture, each well usually generated 2 to 4 × 10^2^ OCs. The dishes then were treated with 0.001% pronase and 0.02% EDTA to remove OBs. More than 99% of the adherent cells prepared were TRACP^+^ and multinucleated OCs.

The human breast adenocarcinoma cell line MCF7 was obtained from American Type Culture Collection (ATCC, Manassas, VA, USA) and maintained in DMEM supplemented with 10% FBS.

### Establishment of transient cell lines

To knock down the levels of ER-α36 expression, primary cultures of OBs and OCs from normal postmenopausal women were plated at a density of 2 × 10^4^ cells/well (OBs) or 2 to 4 × 10^2^ cells/well (OCs) in 24-well plates and transfected 24 hours later with the vector expression shRNA ER-α36 or the empty expression vector (shRNA control) using Lipofectamine 2000 (Invitrogen, Carlsbad, CA, USA) according to the manufacturer's instructions. After 24 hours, cells were transfected again with the expression vector and recovered for another 24 hours and then prepared for the experiment.

To express recombinant ER-α36, primary cultures of OBs and OCs from osteoporotic postmenopausal women were plated at a density of 2 × 10^4^ cells/well (OBs) or 2 to 4 × 10^2^ cells/well (OCs) in 24-well plates and transfected 24 hours later with the ER-α36 expression vector driven by the cytomegalovirus promoter (vector–ER-α36) or the empty expression vector (vector-control) using the FuGene6 transfection reagent (Roche Molecular Biochemicals, Indianapolis, IN, USA). Twenty-four hours after transfection, transfected cells were prepared for experiment.

### Western blot analysis

To detect expression levels of ER-α36 and ER-α66 protein and ERK1/2 phosphorylation, Western blot analysis was performed as described previously.(21,26) Briefly, equal amounts of protein (40 µg/lane) were subjected to SDS gel electrophoresis and transferred to a polyvinylidene difluoride membrane (Amersham Pharmacia Biotech, GE Healthcare Biosciences, Pittsburgh, PA, USA). The membranes were incubated with primary antibodies overnight at 4°C and then incubated with appropriate horseradish peroxidase–conjugated secondary antibodies (1:2500; Santa Cruz Biotechnology) for 1 hour at room temperature. The blots then were visualized with the chemiluminescent detection method using the SuperSignal West Pico Substrate (Pierce, Thermo Fisher Scientific, Rockford, IL, USA).

### Assessment of cell proliferation and apoptosis

OB proliferation was measured using the [^3^H]thymidine assay, as described previously.(27)

Cell apoptosis was measured with TUNEL assay using the in situ cell death detection kit (Roche Molecular Biochemicals, Indianapolis, IN, USA) according to the manufacturer's protocol. The apoptotic and normal cells were examined under the light microscope. Cells with purple nuclei were considered to be apoptotic, and cells with red nuclei were considered to be normal. For each well, 500 OBs or 100 OCs were counted, and the number of apoptotic cells was expressed as the percentage of the total cell population.

### Measurement of ALP activity, osteocalcin secretion and mineralized matrix formation

Alkaline phosphatase (ALP) activity, osteocalcin secretion, and mineralized matrix formation were measured as described previously.(21) Briefly, cells were homogenized, and ALP activity was assayed by spectrophotometric measurement of *p*-nitrophenol release. Osteocalcin released into the culture medium was measured using a specific radioimmunoassay kit (DiaSorin, Inc., Stillwater, MN, USA). Mineralized matrix was examined with alizarin red S staining, and the stained matrix was assessed using a Nikon Diaphot inverted microscope and photographed using a Nikon camera (Nikon, Melville, NY, USA). To normalize protein expression to total cellular protein, an aliquot of the cell lysates was measured with the Bradford protein assay.

### Quantitative real-time PCR (qRT-PCR) analysis

qRT-PCR was performed using a Roche Molecular Light Cycler (Roche Molecular Biochemicals). Total RNA was isolated using the Trizol reagent (Invitrogen), and reverse transcription was performed using 1.0 µg of total RNA and the SuperScript II Kit (Invitrogen). Amplification reactions were set up in 25-µL reaction volumes containing amplification primers and SYBR Green PCR Master Mix (PE Applied Biosystems, Waltham, MA, USA). The PCR primers were as follows: ER-α36 sense, 5'-CCAAGAATGTTCAACCACAACCT-3', and antisense, 5'-GCACGGTTCATTAACATCTTTCTG-3'; ER-α46 sense, 5'-CATTCTCCGGGACTGCGGTA-3', and antisense, 5'-GTACTGGCCAATCTTTCTCTGCC-3'; ER-α66 sense, 5'-AAGAAAGAACAACATCAGCAGTAAAGTC-3', and antisense, 5'-GGGCTATGGCTTGGTTAAACAT-3'; ER-β sense, 5'-GATGCTTTGGTTTGGGTGAT-3', and antisense, 5'-CTTGTTACTCGCATGCCTGA-3'; osteocalcin sense, 5'-GCAGAGTCCAGCAAAGGTG-3', and antisense, 5'-GCTCCCAGCCATTGATACAG-3'; TRACP sense, 5'-CTTTCTACCGCCTGCACTTC-3', and antisense, 5'-GTTTCTTGAGCCAGGACAGC-3'; β-actin sense, 5'-CCCAGCCATGTACGTTGCTA-3', and antisense, 5'-AGGGCATACCCCTCGTAGATG-3'.

To generate a standard curve for quantification, a standard DNA template for each transcript to be assayed was generated by taking the PCR product generated using the primers and diluting this product to generate 10-fold serial dilutions. The absolute mass of each undiluted standard was determined on an Agilent Bioanalyzer (Agilent, Inc., Palo Alto, CA, USA), and copy number was calculated from DNA size. To allow effective comparison of transcript levels between samples, the amount of each transcript is given as copies per 100,000 copies of *β-actin* mRNA. Amplification data were analyzed using the Sequence Detector System Software (PE Applied Biosystems).

### Measurement of reactive oxygen species (ROS) levels

Cells were loaded with 100 µM 2',7'-dichlorodihydrofluorescein diacetate (H_2_ DCFDA) prepared in 1 × PBS for 30 minutes at 37°C, washed, and lysed in 90% DMSO/10% PBS for 10 minutes in the dark. Dichlorofluorescein fluorescence was determined using a fluorescent plate reader (Dynex Technologies, Inc., Chantilly, VA, USA) with 485-nm excitation and 520-nm emission wavelengths.

### Statistical analyses

All calculations were performed using SPSS Version 13.0 for Windows (SPSS, Inc., Chicago, IL, USA). Data are presented as mean ± SD or mean. Comparisons were made using a one-way ANOVA, and differences were considered significant at *p* < .05. The correlations between ER-α36 expression in bone, BMD, and serum bone biochemical markers were analyzed using Pearson's correlation and a partial correlation analysis.

## Results

### ER-α36 is strongly expressed in OBs and OCs from normal postmenopausal women

**Fig. 1:**
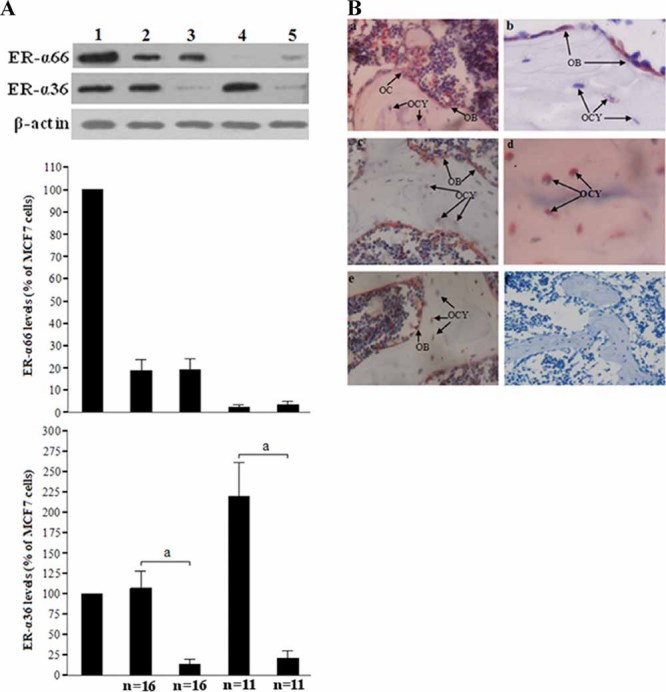
Expression of ER-α36 in bone cells. (*A*) Western blot analysis of ER-α36 and ER-α66 levels in OBs (2) and OCs (4) from normal postmenopausal women and in primary OBs (3) and OCs (5) from osteoporotic postmenopausal women. MCF7 cells (1) were used as a positive control. Shown are representative autoradiographs. The levels of ER-α36, ER-α66, and β-actin were quantitated by densitometric analysis of the autoradiographs, and the ER-α36/β-actin or ER-α66/β-actin ratio in MCF7 cells was arbitrarily set at 100%. The bars represent the mean ± SD. ^a^*p* < .05. *n* represents the number of women in group. (*B*) Immunohistochemical staining for ER-α36 expression in OBs, OCs, and osteocytes (OCY) of human bone sections: vertebral cancellous (*a*, ×200) and cortical (*b*, ×400) bone from a postmenopausal woman; limb cancellous (*c*, ×200) and cortical (*d*, ×400) bone from a postmenopausal woman; limb cancellous bone from a man (*e*, ×200); nonspecific staining in limb cancellous bone from a postmenopausal women as a negative control (*f*, ×100). Shown are representative sections.

Western blot analysis revealed that ER-α36 was strongly expressed in primary OBs and OCs from normal postmenopausal women and weakly expressed in primary OBs and OCs from osteoporotic postmenopausal women (Fig. 1*A*). We also found that expression levels of ER-α66 were similar in primary OBs from normal and osteoporotic postmenopausal women and weakly expressed in primary OCs from normal and osteoporotic postmenopausal women (Fig. 1*A*). Expression of both ER-α36 and ER-α66 proteins was readily detected in an ER^+^ breast cancer cell line MCF7 (Fig. 1*A*).

Immunohistochemistry assay of bone paraffin sections also revealed ER-α36 expression in vertebral and nonvertebral cancellous and cortical bone cells (Fig. 1*B*).

### E_2_ at 100 pM concentrations or greater inhibits ER-α36 expression in primary OBs and OCs from normal postmenopausal women

**Fig. 2:**
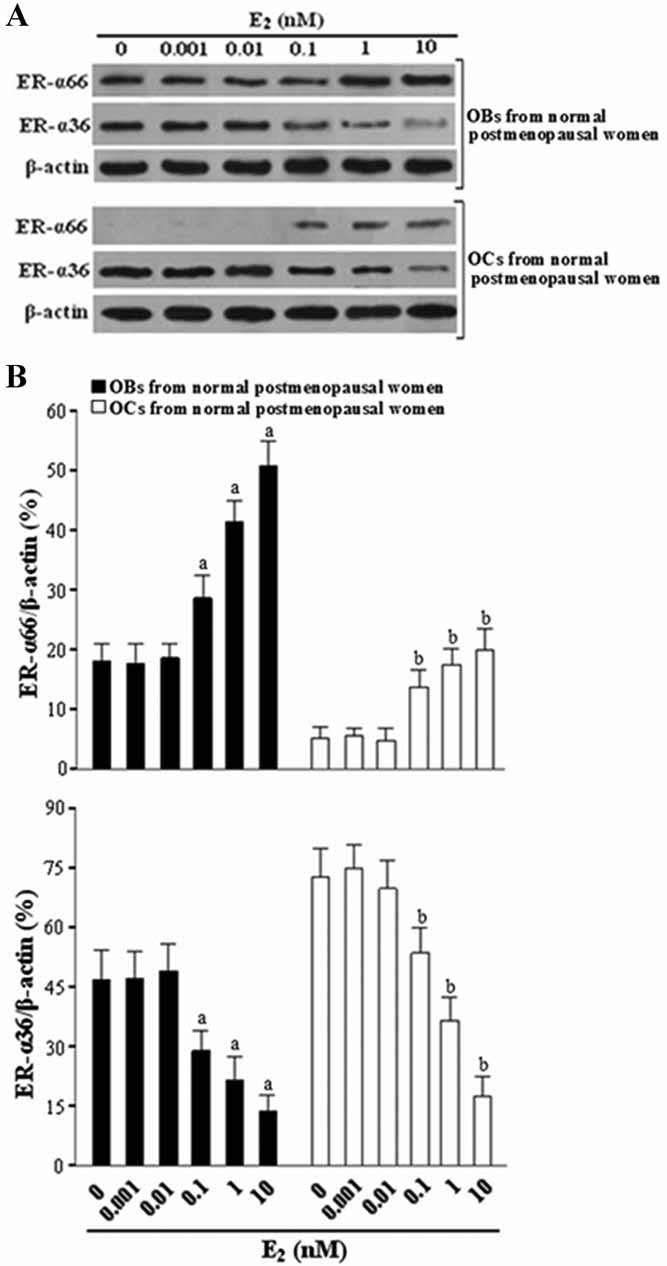
E_2_ at 100 pM or greater concentrations reduces ER-α36 expression and induces ER-α66 expression in OBs and OCs from normal postmenopausal women. Cells were treated with various concentrations of E_2_ for 24 hours and lysed for Western blot analysis. (*A*) Shown are representative autoradiographs. (*B*) ER-α36 and ER-α66 protein expression was expressed as densitometry of ER-α36/β-actin and ER-α36/β-actin. The bars represent the mean ± SD. ^a^*p <* .05 versus vehicle-treated OBs. ^b^*p* < .05 versus vehicle-treated OCs (*n* = 3).

Cells were treated with 1 pM to 10 nM of E_2_ for 24 hours, and the expression levels of ER-α36 and ER-α66 were examined with Western blot analysis. E_2_ induced a significant decrease in the expression levels of ER-α36 in OBs and OCs from normal postmenopausal women in a concentration-dependent manner compared with control cells treated with vehicle, whereas E_2_ concentrations of 10 pM or less failed to alter ER-α36 expression (Fig. 2). In contrast, E_2_ at 100 pM concentrations or more stimulated ER-α66 expression in these cells (Fig. 2).

### ER-α36 mediates postmenopausal low-level E_2_ -induced mitogenic, antiapoptotic, and antiosteogenic effects in OBs and proapoptotic effects in OCs

**Fig. 3:**
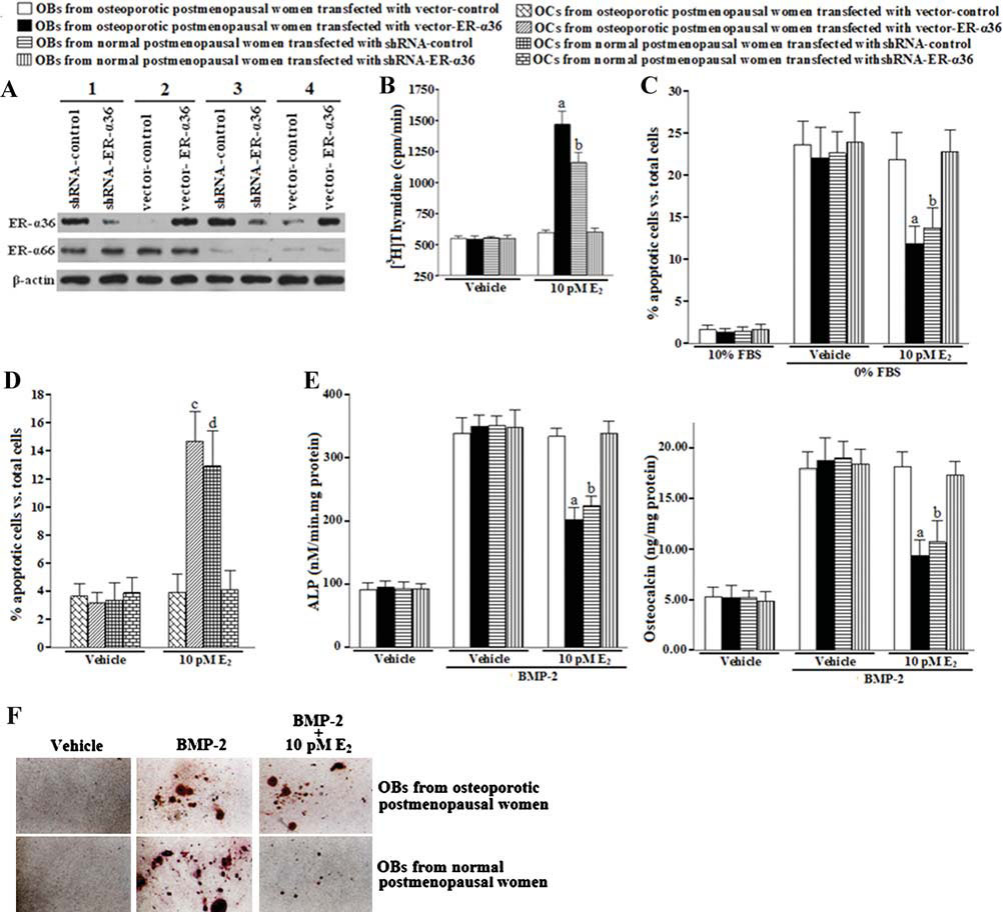
ER-α36 mediates postmenopausal low-level E_2_ -induced mitogenic, antiapoptotic, and antiosteogenic effects in OBs and proapoptotic effects in OCs. (*A*) Western blot analysis of ER-α36 and ER-α66 levels in OBs (1) and OCs (3) from normal postmenopausal women after transfection with the shRNA ER-α36 or the shRNA control and in OBs (2) and OCs (4) from osteoporotic postmenopausal women after transfection with the vector ER-α36 or the vector control. (*B*) ER-α36 mediates E_2_ -induced proliferation of OBs. OBs were treated with vehicle or 10 pM E_2_ for 24 hours. Cell proliferation was determined by measuring [^3^H]thymidine incorporation and expressed as counts per minute. (*C*) ER-α36 mediates E_2_ -induced inhibition of apoptosis in OBs induced by serum deprivation. Serum-deprived OBs were treated with vehicle or 10 pM of E_2_ for 48 hours; 10% FBS–treated cells were used as a control. (*D*) ER-α36 mediates E_2_ -induced apoptosis of OCs. OCs were treated with vehicle or 10 pM of E_2_ for 6 hours. Cell apoptosis was assessed using TUNEL staining and expressed as percentage of TUNEL^+^ cells versus total cells. (*E*) ER-α36 mediates E_2_ -induced inhibition of ALP activity and osteocalcin secretion in OBs induced by BMP-2. OBs were cultured in the medium containing 100 ng/mL of BMP-2 and treated with vehicle or 10 pM E_2_ for 48 hours. Cells were homogenized for ALP activity assay, and cell culture media were collected for osteocalcin secretion assay. (*F*) ER-α36 mediates E_2_ -induced inhibition of matrix mineralization in OBs induced by BMP-2. Shown is a representative microscopic view of the alizarin red S staining in 24-well plates for cells treated with vehicle, BMP-2 (100 ng/mL), or a combination of BMP-2 and 10 pM of E_2_ for 20 days. The bars represent the mean ± SD. ^a^*p* < .05 versus 10 pM E_2_ -treated OBs from osteoporotic postmenopausal women transfected with the vector-control. ^b^*p* < .05 versus 10 pM E_2_ -treated OBs from normal postmenopausal women transfected with the shRNA ER-α36. ^c^*p* < .05 versus 10 pM E_2_ -treated OCs from osteoporotic postmenopausal women transfected with the vector-control. ^d^*p* < .05 versus 10 pM E_2_ -treated OCs from normal postmenopausal women transfected with the shRNA ER-α36 (*n* = 5).

Western blot analysis indicated that primary OBs and OCs from normal postmenopausal women expressed endogenous ER-α36, which could be knocked down by introduction of an ER-α36-specific shRNA, whereas primary OBs and OCs from osteoporotic postmenopausal women expressed undetectable levels of ER-α36, which could be increased with introduction of a recombinant ER-α36 expression vector (Fig. 3*A*).

We found that postmenopausal low-level E_2_ (10 pM) induced proliferation and inhibited apoptosis and differentiation in ER-α36-expressing OBs, induced apoptosis in ER-α36-expressing OCs, but had no effect on the proliferation, apoptosis, or differentiation of ER-α36^−^ cells (Fig. 3*B–F*).

### ER-α36 mediates ERK1/2 activation induced by postmenopausal low-level E_2_ in OBs and OCs

**Fig. 4:**
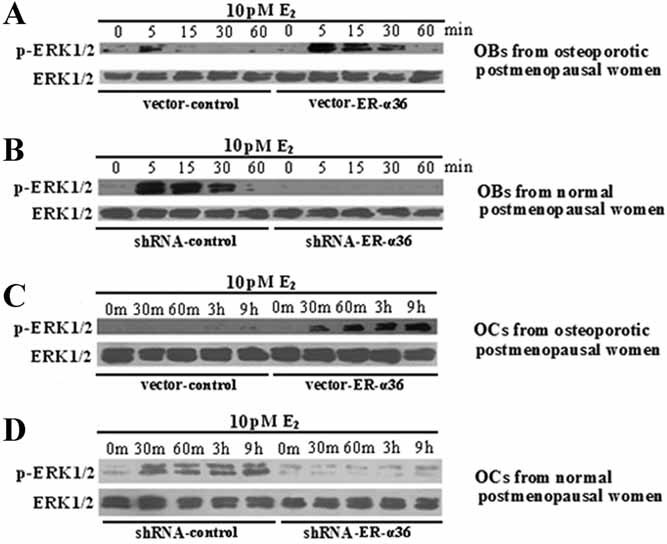
ER-α36 mediates postmenopausal low-level E_2_ -induced transient ERK1/2 pathway activation in OBs and sustained ERK1/2 pathway activation in OCs. (*A*, *B*) E_2_ induces transient ERK1/2 phosphorylation in ER-α36-expressing OBs. OBs from osteoporotic postmenopausal women were transfected with the vector-ER-α36 or the vector-control, and OBs from normal postmenopausal women were transfected with the shRNA ER-α36 or the shRNA control and then treated with 10 pM of E_2_ for 5 to 60 minutes before cell lysis. (*C*, *D*) E_2_ induces sustained ERK1/2 phosphorylation in ER-α36-expressing OCs. OCs from osteoporotic postmenopausal women were transfected with vector-ER-α36 or vector-control, and OCs from normal postmenopausal women were transfected with shRNA ER-α36 or shRNA control and then treated with 10 pM of E_2_ for 30 minutes (*m*) to 9 hours before cell lysis. Cell lysates were analyzed by Western blot. Experiments were repeated three times, and the representative autoradiographs are shown.

Western blot analysis showed that postmenopausal low-level E_2_ (10 pM) induced a dramatic increase of the ERK1/2 phosphorylation within 5 minutes in OBs from normal postmenopausal women that express high levels of endogenous ER-α36 and OBs from osteoporotic postmenopausal women expressing recombinant ER-α36 but not in the OBs barely expressing ER-α36 (OBs from normal postmenopausal women transfected with the ER-α36 shRNA expression vector or OBs from osteoporotic postmenopausal women transfected with empty vector; Fig. 4*A, B*). The increased level of ERK phosphorylation remained for at least 30 minutes before declining to a basal level. We also found that E_2_ stimulated a significant increase of the ERK1/2 phosphorylation in the OCs expressing high levels of endogenous or recombinant ER-α36 but not in the OCs that express undetectable levels of ER-α36 (Fig. 4*C, D*). ERK1/2 phosphorylation in OCs was strong and sustained, lasting for at least 9 hours.

### ROS mediates the prolonged ERK1/2 activation induced by postmenopausal low-level E_2_ in OCs from normal postmenopausal women

**Fig. 5:**
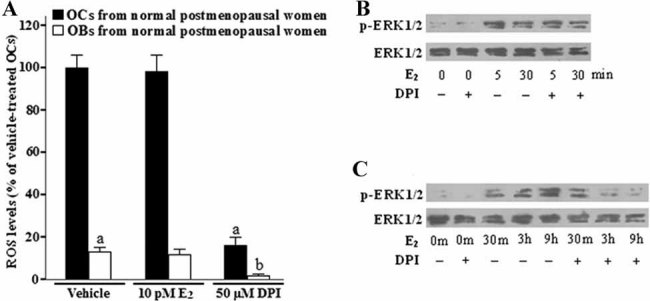
ROS mediates postmenopausal low-level E_2_ -induced sustained ERK1/2 pathway activation in OCs from normal postmenopausal women. (*A*) ROS are strongly expressed in OCs from normal postmenopausal women. OCs and OBs were treated with vehicle or 10 pM of E_2_ for 6 hours or treated with 10 µM of DPI (a ROS inhibitor) for 2 hours. The levels of ROS were examined using a fluorescent probe H_2_ DCFDA. The bars represent the mean ± SD. ^a^*p* < .05 versus vehicle-treated OCs. ^b^*p* < .05 versus vehicle-treated OBs (*n* = 5). (*B*) DPI has no effect on E_2_ -induced transient ERK1/2 phosphorylation in OBs from normal postmenopausal women. Cells were incubated with 10 µM of DPI for 2 hours prior to treatment with 10 pM of E_2_ for 5 or 30 minutes and then lysed for Western blot analysis. (*C*) DPI has no effect on E_2_ -induced transient ERK1/2 phosphorylation but significantly inhibits E_2_ -induced sustained ERK1/2 phosphorylation in OCs from normal postmenopausal women. Cells were incubated with 10 µM of DPI for 2 hours prior to treatment with 10 pM of E_2_ for 30 minutes, 3 hours, or 9 hours and then lysed for Western blot analysis. Experiments were repeated three times, and the representative autoradiographs are shown.

As shown in Fig. 5*A*, OCs from normal postmenopausal women generated large amounts of ROS, whereas OBs from normal postmenopausal women generated much smaller amounts of ROS. E_2_ at 10 pM had no effect on ROS generation in OCs and OBs from normal postmenopausal women. The ROS inhibitor diphenylene iodonium (DPI) significantly inhibited ROS generation in these cells (Fig. 5*A*) but had no effect on E_2_ -induced transient ERK1/2 activation in OBs from normal postmenopausal women (Fig. 5*B*). In addition, DPI had no effect on E_2_ -dependent transient ERK1/2 activation but significantly inhibited E_2_ -induced prolonged ERK1/2 activation in OCs from normal postmenopausal women (Fig. 5*C*).

### ERK1/2 inhibitor blocks postmenopausal low-level E_2_ -induced mitogenic, antiapoptotic, and antiosteogenic effects in OBs and proapoptotic effects in OCs, and the ROS inhibitor blocks E_2_ -induced apoptosis in OCs

**Fig. 6:**
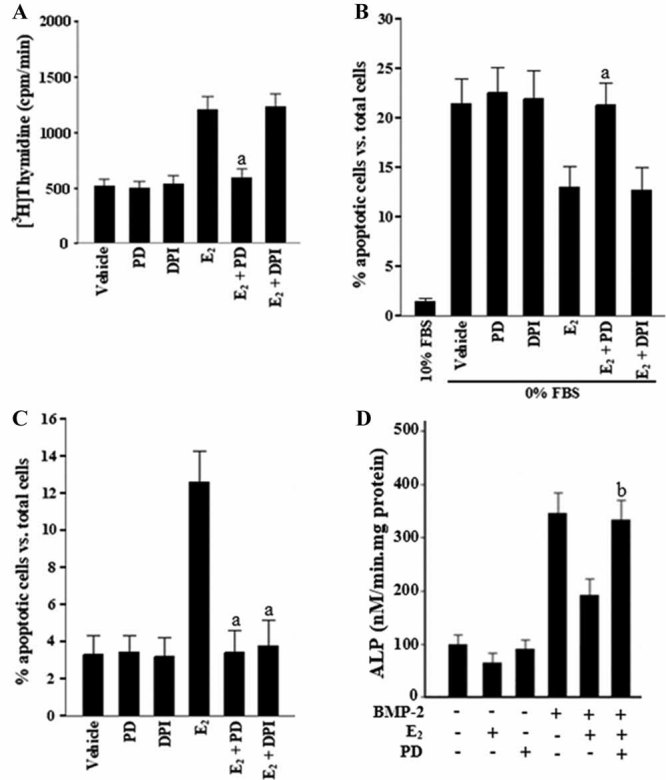
ERK1/2 inhibitor blocks postmenopausal low-level E_2_ -induced mitogenic, antiapoptotic, and antiosteogenic effects in OBs and proapoptotic effects in OCs, and the ROS inhibitor blocks E_2_ -induced apoptosis in OCs. (*A*) ERK1/2 inhibitor abolishes E_2_ -induced proliferation of OBs from normal postmenopausal women. Cells were incubated with ERK1/2 inhibitor PD98059 (PD; 10 µM) or ROS inhibitor DPI (10 µM) for 2 hours prior to treatment with 10 pM of E_2_ for 24 hours. Cell proliferation was determined by measuring [^3^H]thymidine incorporation and expressed as counts per minute. (*B*) ERK1/2 inhibitor abolishes E_2_ -induced inhibition of apoptosis in OBs from normal postmenopausal women. Serum-deprived cells were incubated with 10 µM of PD or 10 µM of DPI for 2 hours prior to treatment with 10 pM of E_2_ for 48 hours; 10% FBS-treated cells were used as control. (*C*) ROS or ERK1/2 inhibitor abrogates E_2_ -induced apoptosis of OCs from normal postmenopausal women. Cells were incubated with 10 µM of PD or 10 µM of DPI for 2 hours prior to treatment with 10 pM of E_2_ for 6 hours. Apoptosis was assessed using TUNEL staining and expressed as percentage of TUNEL^+^ cells versus total cells. (*D*) ERK1/2 inhibitor abolishes E_2_ -induced inhibition of ALP activity in OBs from normal postmenopausal women. BMP-2-treated cells were incubated with 10 µM of PD for 2 hours prior to treatment with 10 pM of E_2_ for 48 hours and then homogenized for ALP activity assay. The bars represent the mean ± SD. ^a^*p* < .05 versus 10 pM E_2_ -treated cells. ^b^*p* < .05 versus BMP-2 + E_2_ -treated cells (*n* = 5).

As shown in Fig. 6*A*, OBs from normal postmenopausal women exposed to postmenopausal low-level E_2_ (10 pM) alone exhibited a marked increase in [^3^H]thymidine incorporation, whereas OBs pretreated with the ERK1/2 inhibitor PD98059 completely abolished [^3^H]thymidine incorporation induced by E_2_ . These OBs exhibited no change in E_2_ -induced [^3^H]thymidine incorporation after treatment with the ROS inhibitor DPI.

Figure 6*B* shows that serum-deprived OBs from normal postmenopausal women exposed to 10 pM of E_2_ alone exhibited fewer number of TUNEL^+^ cells than control OBs treated with vehicle, whereas serum-deprived OBs pretreated with PD98059 exhibited an increase of the number of TUNEL^+^ cells and serum-deprived OBs pretreated with DPI exhibited no change in the number of TUNEL^+^ cells.

We also noticed a significant increase in the number of TUNEL^+^ OCs from normal postmenopausal women treated with 10 pM of E_2_ and a virtual lack of the TUNEL^+^ cells when OCs were pretreated with DPI or PD98059, respectively (Fig. 6*C*).

In addition, we found that PD98059 treatment reversed the inhibitory effect of 10 pM of E_2_ on bone morphogenetic protein 2 (BMP-2)–induced ALP activity in OBs from normal postmenopausal women (Fig. 6*D*).

### ER-α36 mediates postmenopausal low-level E_2_ -induced suppression of bone turnover in cultured bone tissues in vitro

**Fig. 7:**
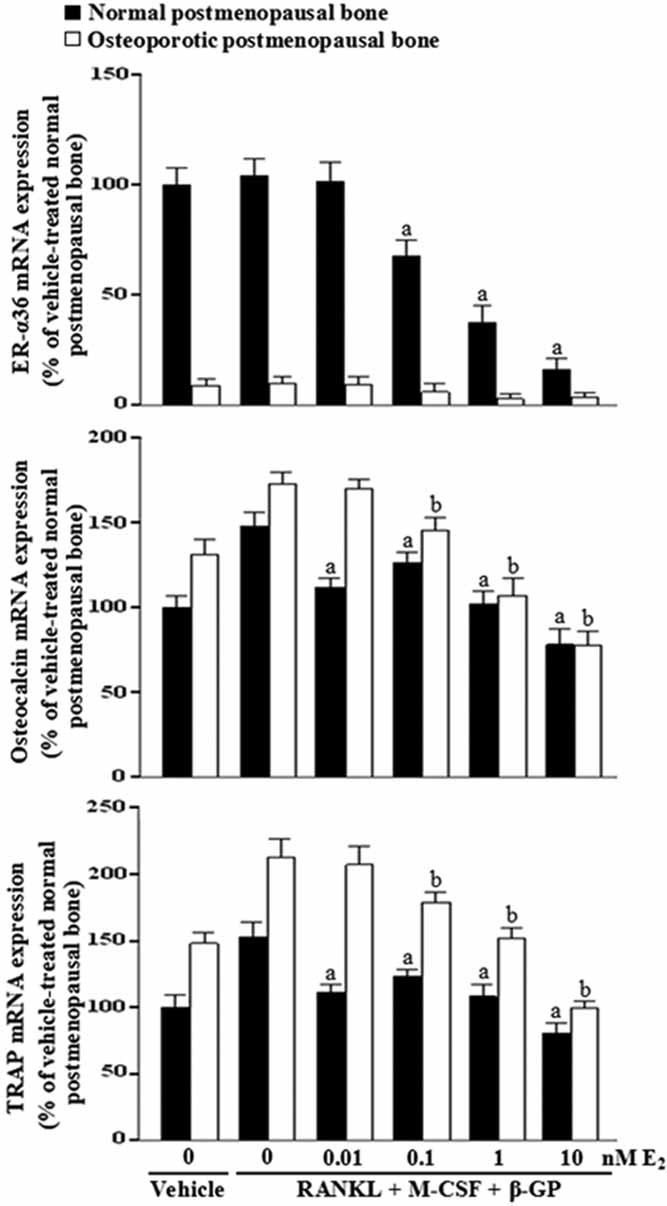
ER-α36 mediates postmenopausal low-level E_2_ -induced inhibition of bone resorption and formation in cultured bone tissues. Normal or osteoporotic postmenopausal bone tissues were cultured in a medium containing 10% FBS and treated with vehicle, bone turnover–stimulating factors (50 ng/mL RANKL + 25 ng/mL M-CSF + 10 mM β-GP), or bone turnover–stimulating factors with various concentrations of E_2_ for 28 days. mRNA levels of ER-α36, osteocalcin, and TRACP were determined using qRT-PCR and given as fold induction relative to vehicle-treated normal postmenopausal bone. The bars represent the mean ± SD. ^a^*p* < .05 versus RANKL + M-CSF + β-GP–treated normal postmenopausal bone (*n* = 5).

As shown in Fig. 7, incubation with bone turnover–stimulating medium [containing RANKL + monocyte colony-stimulating factor (M-CSF) + β-glycerophosphate (β-GP)] led to a significant increase in the mRNA expression levels of the bone-resorption marker tartrate-resistant acid phosphatase (TRACP) and the bone-formation marker osteocalcin in cultured bone tissues from normal and osteoporotic postmenopausal women. Cotreatment with postmenopausal low-level E_2_ (10 pM) significantly inhibited the induction of TRACP and osteocalcin in normal postmenopausal bone tissues that express high levels of endogenous ER-α36. However, in osteoporotic postmenopausal bone tissues that express low levels of endogenous ER-α36, the effects of 10 pM of E_2_ were reduced dramatically. Cotreatment with increasing doses of E_2_ (100 pM to 10 nM) inhibited ER-α36 expression and suppressed the induction of TRACP and osteocalcin in both normal and osteoporotic postmenopausal bone tissues.

### Characteristics of the study population

Table 1 shows a comparison of baseline values for the clinical characteristics of the premenopausal women and normal, osteopenic, and osteoporotic postmenopausal women. The four groups were preselected for matched body weight. All postmenopausal groups did not vary significantly with respect to age or years since menopause. More important, they were selected successfully on the basis of having similar levels of serum E_2_ and FEI. As expected, we found that the serum bone-formation marker osteocalcin and the serum bone-resorption marker NTX were expressed at higher levels (*p* < .05) in the osteoporotic or osteopenic postmenopausal groups than those in the normal postmenopausal group or the premenopausal group.

**Table 1:** Characteristics of the Study Population

		Postmenopausal		
		
	Premenopausal	Normal	Osteopenic	Osteoporotic
*n*	60	33	31	30
Age (yr)	43	56	57	57
YSM(yr)	0	7	8	8
Body weight (kg)	57	57	57	56
E_3_ (pM)		20.8	20.0	19.6
SHBG (nM)		57.9	58.4	60.3
FEI		0.45	0.45	0.43
Total hip BMD (g/cm^2^)	0.855	0.805	0.714^a^,^b^	0.586^a^,^b^,^c^
L1-L4 BMD (g/cm^3^)	0.749	0.730	0.626^a^,^b^	0.538^a^,^b^,^c^
Serum osteocalcin (ng/ml)	5.66	6.62	9.07^a^,^b^	13.80^a^,^b^,^c^
Serum NTX (nMBCE)	14.S	14.8	22 8^a^,^b^	31.0^a^,^b^,^c^
Copies ER-α36 mRNA per 100,000 copies β-actin	253	3729^a^	1253^a^,^b^	554^b^,^c^
Copies ER-α46 mRNA per 100,000 copies β-actin	1905	803^a^	795^a^	758^a^
Copies *ER-*α*66* mRNAper 100,000 copies β-actin	1971	652^a^	711^a^	730^a^
Copies ER-βmRNA per 100,000 copies β-actin	911	572	459	586

Data are means. YSM = years since menopause; BCE = bone collagen equivalents.

a *p* < .05 versus premenopause group.

b *p* < .05 versus normal postmenopausal group.

c *p* < .05 versus osteopenic postmenopausal group.

Table 1 also includes the levels (copies per 100,000 copies of β-actin) of *ERα36*, *ERα46*, *ERα66*, and *ERβ* mRNA expression in bone biopsies, as determined by qRT-PCR. In premenopausal women, the levels of ER-α36 expression were lower than the levels of ER-α46, ER-α66, and ER-β expression in bone. In all postmenopausal groups, however, there was a significant decrease of ER-α46 and ER-α66 levels in bone and a non-significant decrease in the ER-β level compared with the premenopausal group. There was no significant difference in mRNA levels of *ERα46*, *ERα66*, and *ERβ* expression among all postmenopausal groups. Remarkably, the mRNA levels of *ERα36* in the normal postmenopausal group were significantly higher (*p* < 0.05) than in the other two postmenopausal groups (osteoporotic or osteopenic) and the premenopausal group.

### *ERα36* mRNA expression is positively associated with BMD and negatively associated with bone biochemical markers

The correlation coefficients between the mRNA levels of *ERα36* and both BMD and the bone biochemical markers are shown in Tables 2 and 3. In postmenopausal women, a significant positive relationship was observed between the levels of ER-α36 expression in bone and BMD of the lumbar spine and total hip. However, a significantly negative relationship was observed between the levels of ER-α36 expression in bone and the serum bone-formation marker osteocalcin and the serum bone-resorption marker NTX. The correlations still remained significant after adjustment for age and fat mass. No significant correlations, however, were observed between the levels of ER-α36 expression in bone and BMD and serum bone biochemical markers in premenopausal women, and no significant correlations were found between the levels of ER-α46, ER-α66, and ER-β expression in bone with BMD and serum bone biochemical markers in both pre- and postmenopausal women.

**Table 2:** Correlation Coefficients (*r*) of mRNA Levels of ER-α36, ER-α46, ER-α66, and ER-β in Bone and BMD in Premenopausal and Postmenopausal Women

	Premenopausal women (n = 60)				Postmenopausal women (n = 94)			
		
	L1-L4 BMD		Total hip BMD		L1-L4 BMD		Total hip BMD	
				
	Unadjusted	Adjusted	Unadjusted	Adjusted	Unadjusted	Adjusted	Unadjusted	Adjusted
ER-α36	0.040	0.037	0.029	0.025	0.305^a^	0.283^a^	0.273^a^	0.248^a^
ER-α46	0.030	0.024	0.045	0.041	0.027	0.026	0.029	0.024
ER-α66	0.057	0.052	0.050	0.044	0.036	0.029	0.035	0.031
ER-β	−0.036	−0.032	−0.035	−0.026	0.042	0.032	0.039	0.034

*Note:* Pearson's correlation coefficients and partial correlation coefficients after adjustment for age and fat mass are shown.

a *p* < .05.

**Table 3:** Correlation Coefficients (*r*) of mRNA Levels of ER-α36, ER-α46, ER-α66, and ER-β in Bone With Bone Biochemical Markers in Premenopausal and Postmenopausal Women

	Premenopausal women (n = 60)				Postmenopausal women (n = 94)			
		
	Serum osteocalcin		Serum NTX		Serum osteocalcin		Serum NTX	
				
	Unadjusted	Adjusted	Unadjusted	Adjusted	Unadjusted	Adjusted	Unadjusted	Adjusted
ER-α36	0.041	0.036	−0.033	−0.027	−0.253^a^	−0.220^a^	−0.356^a^	−0.329^a^
ER-α46	−0.031	−0.028	−0.035	−0.029	−0.036	−0.029	−0.037	−0.026
ER-α66	−0.067	−0.064	−0.083	−0.074	−0.037	−0.031	−0.048	−0.038
ER-β	0.053	0.045	−0.041	−0.032	0.040	0.029	0.043	0.032

*Note:* Pearson's correlation coefficients and partial correlation coefficients after adjustment for age and fat mass are shown.

a *p* < .05.

## Discussion

In this study, we found that ER-α36 is a critical estrogen receptor responsible for beneficial E_2_ signaling in bone metabolism of postmenopausal women. we examined ER-α36 expression in primary bone cells and found that ER-α36 was highly expressed in OBs and OCs from normal postmenopausal women and weakly expressed in OBs and OCs from osteoporotic postmenopausal women. Recently, we reported that ER^−^ breast cancer cells expressing endogenous ER-α36 and ER^+^ breast cancer cells and ER^−^ HEK293 cells expressing high levels of recombinant ER-α36 responded to a very low concentration of E_2_ : activation of the MAPK/ERK signaling pathway at the picomolar range, suggesting that cells expressing high levels of ER-α36 are hypersensitive to E_2_ .(18) Based on these results, we suggest that ER-α36 probably plays an important role in regulating bone metabolism at the low E_2_ levels of the postmenopausal women.

It is well known that aged bone is gradually replaced by new bone tissue through a process called *bone remodeling* or *bone turnover*. Bone remodeling occurs through the coordinated actions of bone-forming OBs and bone-resorbing OCs. An augmented loss of the OBs and reduced apoptosis of the OCs in menopausal women could account for the focal imbalance between bone resorption and formation after a dramatic reduction in E_2_ concentration.(28,29) Here we found that OBs and OCs from normal postmenopausal women express high levels of endogenous ER-α36 and response to E_2_ at a concentration of 10 pM (a postmenopausal level of E_2_ ). The same concentration of E_2_ produced significant mitogenic and antiapoptotic effects on OBs and a proapoptotic effect on OCs. In addition, shRNA knockdown experiments demonstrated that ER-α36 mediates these activities in OBs and OCs. In primary cultures of OBs and OCs from osteoporotic postmenopausal women that express undetectable levels of endogenous ER-α36, E_2_ at a level of 10 pM had no effects on cell proliferation or apoptosis. However, these activities were restored when a recombinant ER-α36 expression vector was introduced into these cells. These data thus demonstrated that ER-α36 can mediate mitogenic and antiapoptotic signaling induced by the postmenopausal level of E_2_ in OBs and proapoptotic signaling in OCs. Furthermore, we showed here that ER-α36 also can mediate antiosteogenic signaling induced by the postmenopausal level of E_2_ in OBs. This enhanced sensitivity of bone cells to E_2_ is consistent with the recent reports from other laboratories that low concentrations of estrogens were able to trigger nongenomic actions in pituitary cells and pancreatic beta cells.(30–32)

Our previous study indicated that ER-α36 mediates E_2_ -induced rapid activation of the MAPK/ERK pathway.(13) Lower concentrations of estrogen have been found to potently exert nongenomic actions in breast cancer cells, pituitary cells, and pancreatic beta cells.(18,30–32)

Our data demonstrated that ER-α36 mediated a rapid and transient activation of the MAPK/ERK pathway in OBs and a strong and prolonged activation of the MAPK/ERK pathway in OCs induced by postmenopausal low-level E_2_ (10 pM). In general, rapid and transient ERK activation can enhance cell survival and stimulate proliferation.(33) However, a prolonged or sustained ERK activation (ERK phosphorylation is maintained for more than 6 hours) triggers an apoptotic signal.(34) One of the underlying mechanisms of the prolonged ERK activation is generation of the ROS.(34) OCs usually express high amounts of TRACP that can generate ROS through its redox-active iron via a Fenton reaction.(35) We found that OCs from normal postmenopausal women generated large amounts of ROS, whereas OBs from normal postmenopausal women produced much smaller amounts of ROS. Inhibition of ROS had no effect on 10 pM E_2_ -induced transient ERK1/2 activation in OBs. In OCs, however, inhibition of ROS significantly abrogated 10 pM E_2_ -induced prolonged ERK1/2 activation but had no effect on E_2_ -induced transient ERK1/2 activation. These results suggest that the postmenopausal level of E_2_ -induced transient ERK activation in both OBs and OCs is mediated by ER-α36, whereas the sustained and prolonged ERK activation in OCs is mediated by both ER-α36 and ROS. Our results thus extend the underlying mechanisms involved in E_2_ -dependent sustained ERK activation in OCs.

Furthermore, the inhibition of ERK also attenuated the postmenopausal level of E_2_ -induced mitogenic, antiapoptotic, and antiosteogenic effects in OBs from normal postmenopausal women and the proapoptotic effects in OCs from normal postmenopausal women and the inhibition of ROS-attenuated E_2_ activities in OCs but not in OBs. We thereby concluded that ER-α36 mediated the effects of postmenopausal low-level E_2_ on proliferation, apoptosis, and differentiation of OBs through transient activation of the MAPK/ERK and that ER-α36 mediated the postmenopausal low-level E_2_ -mediated proapoptotic activity of OCs through prolonged activation of the MAPK/ERK pathway with the involvement of ROS. The MAPK/ERK-dependent inhibition of OB differentiation by E_2_ found here is consistent with a previous report that E_2_ attenuates differentiation of primary cultures of calvaria- or bone marrow–derived OBs via activation of ERK.(36)

We also found that the postmenopausal level of E_2_ (10 pM) significantly inhibited bone-turnover-stimulating medium (containing RANKL + M-CSF + β-GP)–induced mRNA expression levels of the bone resorption marker TRACP and the bone-formation marker osteocalcin in normal postmenopausal bone tissues that express high levels of endogenous ER-α36. However, in osteoporotic postmenopausal bone tissues that express low levels of endogenous ER-α36, E_2_ functions were reduced dramatically. Notably, cotreatment with increasing doses of estrogen (≥10 pM) inhibited ER-α36 expression and suppressed the induction of TRACP and osteocalcin in both normal and osteoporotic bone tissues. We thus postulated that ER-α36 mediates low-level E_2_ -induced suppression of bone turnover in postmenopausal women, whereas the effect of ER-α36 is diminished at higher concentration of E_2_, suggesting that ER-α36 plays an important role in maintenance of normal bone density in postmenopausal women.

We found that the levels of ER-α36 expression were lower than the levels of ER-α46, ER-α66, and ER-β expression in the bone of premenopausal women. In all postmenopausal groups, however, there was a decrease in ER-α46, ER-α66, and ER-β expression levels in bone compared with the premenopausal group. This is in a good agreement with previous reports that a normal estrogen level maintains normal expression of ERs (ER-α66 and ER-β) in various tissues and that estrogen deficiency would lead to a downregulation of their expression.(37) Consistent with these findings, our in vitro results indicated that E_2_ at 100 pM or greater concentrations (equivalent to or exceeding premenopausal serum levels of E_2_ ) stimulates ER-α66 expression and inhibits ER-α36 expression in primary cultures of OBs and OCs from normal postmenopausal women, suggesting that E_2_ regulates its receptor variants differently. However, we did not observe significant changes among mRNA levels of ER-α46, ER-α66, and ER-β in all postmenopausal groups. Remarkably, the mRNA levels of ER-α36 in the normal postmenopausal group were significantly higher than those of the other two postmenopausal groups (osteoporotic or osteopenic) and the premenopausal group. In addition, we found that the levels of *ERα36* mRNA in bone were positively associated with BMD and negatively associated with the serum bone-resorption marker NTX and the serum bone-formation marker osteocalcin in the postmenopausal women but not in the premenopausal women. No significant correlations were found between the mRNA levels of ER-α46, ER-α66, and ER-β with BMD and bone biochemical markers in both pre- and postmenopausal women. Together our results indicate that ER-α36 plays a more important role in regulating bone metabolism than ER-α66, ER-α46, and ER-β in postmenopausal women.

The ER-α36 transcript is initiated from a promoter located in the first intron of the *ERα66* gene,(38) indicating that ER-α36 expression is subjected to a transcription regulation different from ER-α66 and consistent with the finding that ER-α36 is expressed in ER^−^ breast cancer cells that lack ER-α66 expression.(13,14) We demonstrated that G protein–coupled receptor (GPCR) 30 signals through the Src/MAPK/AP-1 pathway to activate the promoter activity and to induce ER-α36 but not ER-α66 expression,(18) suggesting that other GPCR and growth factor signaling pathways may regulate ER-α36 expression. Thus ER-α36 expression is regulated differently in bone cells from normal and osteoporotic postmenopausal women.

Based on our findings, it is possible that OCs of normal postmenopausal women that express high level of endogenous ER-α36 respond to the postmenopausal level of E_2_ by prolonged activation of the MAPK/ERK signaling pathway and undergoing apoptosis, which leads to a suppression of bone resorption. On the other hand, E_2_ also exerts mitogenic and antiapoptotic effects on OBs of normal postmenopausal women who express high levels of ER-α36, which, however, are not translated into an increase in bone formation because osteoblastic differentiation has been suppressed; in addition, it is also possible that bone formation fails to happen without bone resorption because bone formation occurs only at sites of previous resorption during bone remodeling.(39)

Although it is well established that E_2_ treatment prevents bone loss in postmenopausal women, previous studies that looked at the role of residual endogenous estrogens in maintaining bone mass remain controversial.(40–47) Some studies showed that residual E_2_ plays a role in maintaining bone mass and reducing fracture risk in the postmenopausal women.(40–43) Other studies, however, have failed to show a relationship between endogenous E_2_ and bone loss,(44–46) and no clear correlation has been found between endogenous E_2_ levels and bone turnover markers.(47) Here we examined ER-α36 expression in groups of carefully selected osteoporotic, osteopenic, and normal postmenopausal women matched for endogenous levels of serum E_2_ and FEI to eliminate the potential interference of residual E_2_ difference. Our results strongly suggest that ER-α36 expression may be used as a reliable marker to predict residual E_2_ function in bone mass.

Riggs and colleagues(46) hypothesized that the greater bone loss in postmenopausal osteoporosis is the result of impaired responsiveness of bone to postmenopausal levels of sex steroids and ascribed the impaired bone responsiveness to the polymorphisms of the genes for the estrogen receptors or the postreceptor estrogen signaling pathway.(46) In this study we clearly demonstrated that the bone cells from osteoporotic postmenopausal women expressed much lower levels of ER-α36 than bone cells from normal postmenopausal women. We also demonstrated that ER-α36 effectively mediates the bone-sparing effects of residual levels of E_2_ found in postmenopausal women. Thus, based on the findings that high levels of ER-α36 expression and relatively low levels of ER-α66, ER-α46, and ER-β expression in bone of normal postmenopausal women, we conclude that ER-α36 is a critical player among ERs that is involved in beneficial E_2_ signaling in the bone metabolism observed in postmenopausal women.

This study unequivocally demonstrates for the first time that ER-α36 enhances the responsiveness of both OBs and OCs from postmenopausal women to postmenopausal level of E_2_ and that its expression in bone is positively associated with BMD and negatively associated with serum levels of the bone biochemical markers NTX and osteocalcin. Thus we conclude that ER-α36 mediates a bone-sparing effect of the low level of E_2_ in postmenopausal women, and this may guide us to search for novel antiosteoporotic agents that possess the preservative effect on bone via ER-α36 but without the universal side effects on reproductive tissues seen with the original ER-α66.
